# Transesophageal echocardiography-guided implantation of totally implantable venous access devices via the internal jugular vein: retrospective analysis of 297 cases in pediatric patients

**DOI:** 10.1186/s12957-022-02734-8

**Published:** 2022-08-30

**Authors:** Yuanzhen Chen, Dajun Xing, Lixin Wu, Huatian Lin, Ting Lin, Fang Ding, Liang Xu

**Affiliations:** Department of Anesthesiology, Shenzhen Children’s Hospital, Shenzhen, 518000 Guangdong China

**Keywords:** Totally implantable venous access device (TIVAD), Transesophageal echocardiography (TEE), Complication, Catheter tip position, Chest radiography

## Abstract

**Background:**

Accurately positioning totally implantable venous access device (TIVAD) catheters and reducing complications in pediatric patients are important and challenging. A number of studies have shown methods for locating the tip of the TIVAD catheter. We assessed the success and complications of TIVAD implantation guided by transesophageal echocardiography (TEE) via the internal jugular vein (IJV) for 294 patients in this retrospective study.

**Methods:**

From May 2019 to March 2021, 297 cases of TIVADs in our hospital were analyzed in this observational, non-randomized, single-center study. The position of the catheter tip under TEE and chest radiography and rates of periprocedural, early, and late complications were evaluated.

**Results:**

The implantation was successful in 242 (82.3%) cases which was in a proper position, and the results were consistent with those of postoperative chest radiography. A total of 72 complications were recorded. Of these, 1 case had a perioperative complication, 66 had early complications, and 5 had late complications after port implantation. The most common complications were local infection and catheter malposition, namely 10 (13.9%) cases of incision infection and 58 (80.6%) cases of catheter malposition. In total, 6 (8.3%) cases of port explantation were required.

**Conclusion:**

Confirmation of proper TIVAD catheter positioning by TEE through an internal jugular approach in children was accurate and safe.

## Background

Totally implantable venous access devices (TIVADs) have been widely used in patients who need long-term chemotherapy or parenteral nutrition [1, 2]. Demands for TIVADs in cancer patients, especially in children with cancer, are increasing rapidly in recent years [3]. Yazıcı et al. confirmed the importance of TIVADs in children suffering from cancer [4]. Because the peripheral blood vessels of children are relatively thin and fragile, chemotherapy drugs cause certain damage to the blood vessels and physical pain, and drug extravasation leads to local tissue necrosis [5, 6]; the application and accurate position of TIVADs in pediatric patients are of great importance and full of challenges.

Various techniques were used to improve the accurate positioning of TIVADs, such as chest radiography [7], modified surface measurement [8], intravenous electrocardiography [9], transesophageal echocardiography (TEE) [10], and all kinds of depth calculation formulas [11]. In recent years, the application of intraoperative TEE real-time positioning has been introduced [12] and showed obvious advantages, such as low cost and non-radiation. In Shenzhen Children’s Hospital, China, TIVADs have been implanted by interventional radiologists for the last few years. However, multiple radiation exposure has potential adverse effects in pediatric patients [13], especially those suffering from cancer and hematological diseases with extremely few leukocytes. In addition, the cost of interventional guidance was significantly higher than that of TEE. Generally, the average cost of interventional guidance is about 11,000 Ren Min Bi (RMB), while the average cost of TEE guidance is only about 6600 RMB. A prior study confirmed the accuracy of TEE positioning for TIVADs through a left subclavian approach among 36 children [12], but an investigation with a larger sample size is needed to further verify the positive role of TEE.

In this study, we tried a TEE-guided technique for the implantation of TIVADs via an internal jugular access in 294 patients with cancer and other diseases and observed the positions of catheter tips and complications of TIVAD implantation.

## Methods

### Patients

This observational, non-randomized, single-center study assessed the results of 297 TIVADs consecutively placed in 294 pediatric patients with cancer and other diseases in Shenzhen Children’s Hospital, China, from May 2019 to March 2021. Written informed consent of legal guardians regarding surgery and anesthesia was obtained before the procedures. All our procedures consisted of 3 parts: implementation of anesthesia, operation and monitoring of TEE, and implantation of TIVADs. Our team included 4 anesthesiologists, 3 TEE operators, and 4 surgeons with more than 3 years of experience, all of whom have undergone unified training and followed the standard operating procedures (SOPs). This study was approved by the Institutional Review Board for Human Studies in Shenzhen Children’s Hospital, China.

The following are the inclusion criteria:

1. Pediatric patients (age < 18 years)
2. American Society of Anesthesiologists physical status grades I–III
3. Undergoing TIVAD implantation

The following are the exclusion criteria:

1. Diagnosed as coagulopathy
2. Infection in surgical areas

### Anesthesia process

Standardized general anesthesia with endotracheal intubation was performed by 4 anesthesiologists. Anesthetics of induction: propofol 2–3 mg/kg, remifentanyl 2 μg/kg, and rocuronium 0.6 mg/kg. Ventilation after the endotracheal intubation: tidal volume (VT) 8–10 ml/kg, respiratory rate (RR) 16–24 bpm, and keeping end-tidal CO_2_ (ETCO_2_) 30–40 mmHg. Anesthesia maintenance: 2–3% sevoflurane and remifentanyl 0.2 μg/kg/min, adjusted as needed.

Before the operation, use 0.25% ropivacaine about 1 cm below the collarbone for local anesthesia in the operation area to ensure intraoperative and postoperative comfort.

### Surgical procedure

**Fig. 1:**
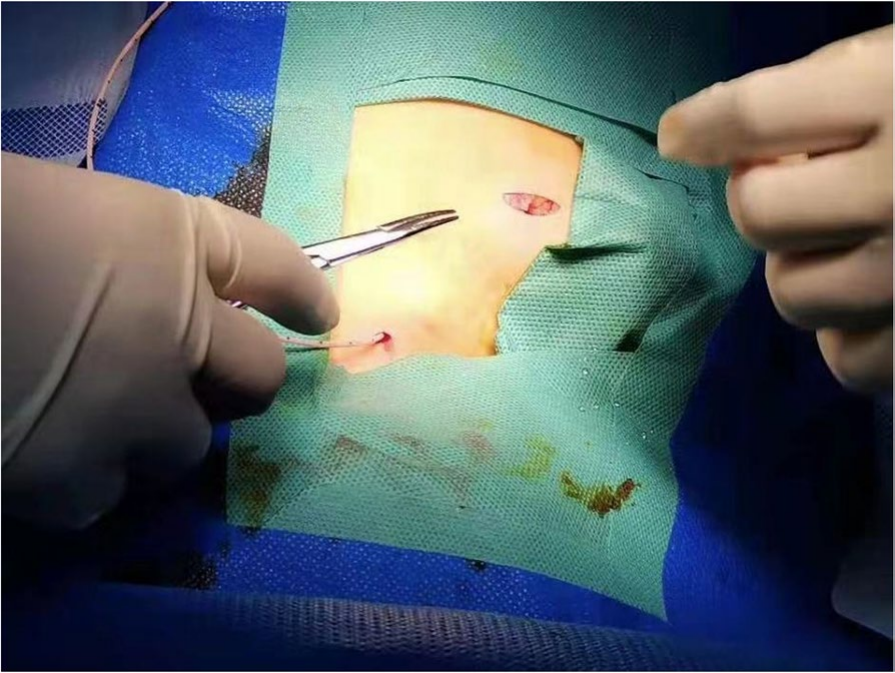
Intraoperative incision images: small incision for internal jugular vein catheter implantation and ipsilateral subclavian port implantation

**Fig. 2:**
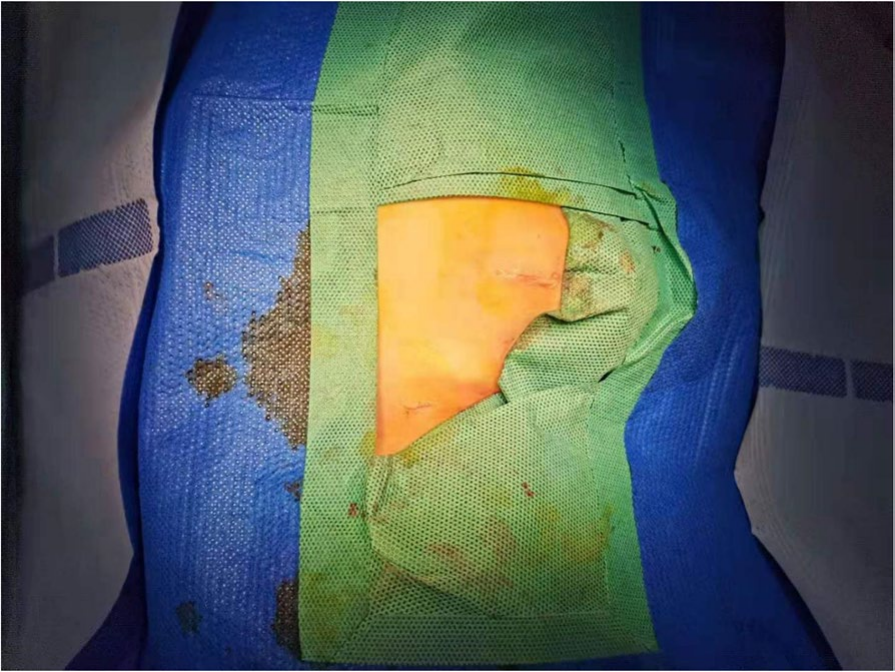
Successful implantation of the TIVAD. TIVAD, totally implantable venous access device

**Fig. 3:**
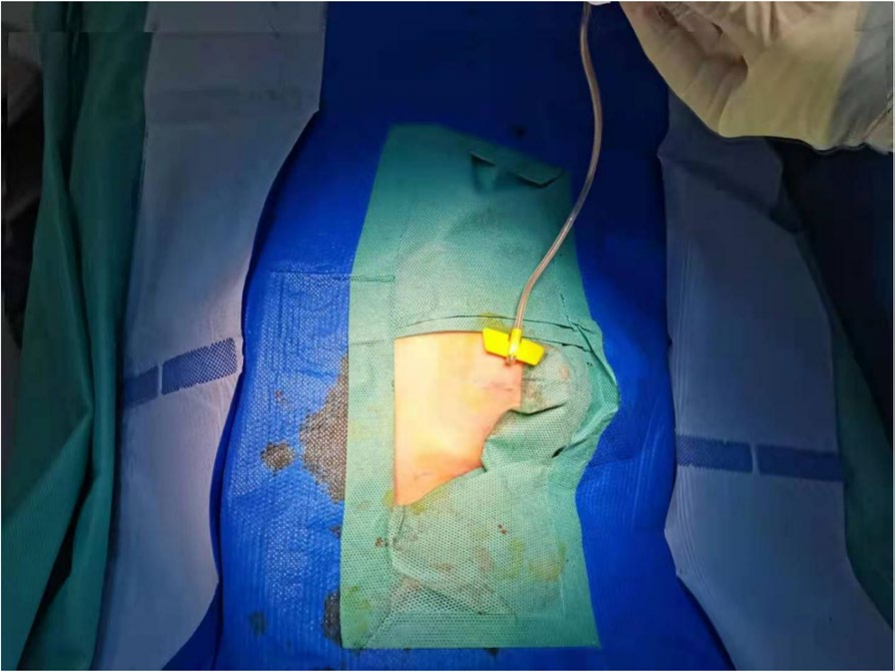
After the port was inserted, a paper clip was inserted into the port, and blood was extracted to ensure successful implantation

Ports were implanted by 4 surgeons who had been trained to follow a standardized implantation procedure. The patient lay in the 5–10° Trendlenburg position, and electrocardiogram (ECG), pulse oximetry, and blood pressure were monitored. The surgical areas of the neck, chest, and shoulder were prepped and draped. The port and supplies were rinsed in 5 U/ml heparin saline. Local anesthesia was applied to the surgical areas with 0.25% ropivacaine for intra- and postoperative comfort about 1 cm under the clavicle before the surgery. Then, the right/left internal jugular vein (IJV) was punctured under ultrasound guidance at a suitable site. A guide wire was inserted into the right/left IJV right after a successful puncture. After small incision formation and vascular expansion at the puncture point, a catheter entered the right/left IJV at approximately 10 cm. The length from the puncture site to the superior vena cava-right atrial (SVC-RA) junction (L1) was determined under TEE guidance. A port chamber was made on the ipsilateral chest under the clavicle for port implantation (Fig. 1). Next, a subcutaneous tunnel was made, from the top of the port chamber to the puncture site. Then, the catheter was pulled from the puncture site to the port chamber through the tunnel, and the length of the tunnel (L2) was measured using a scale on the catheter. The catheter was cut according to the expected length (L1 + L2) and then connected to the port, after the right position of the catheter tip was retested using TEE before the port was fixed and the chamber closed (Fig. 2). Subsequently, the needle of chemotherapy was inserted into the port, and blood was extracted to ensure successful implantation (Fig. 3). The anesthesia would be stopped right after the surgery, and the patient should be escorted to a post-anesthesia care unit (PACU) after recovering from anesthesia.

### Operation with TEE

**Fig. 4:**
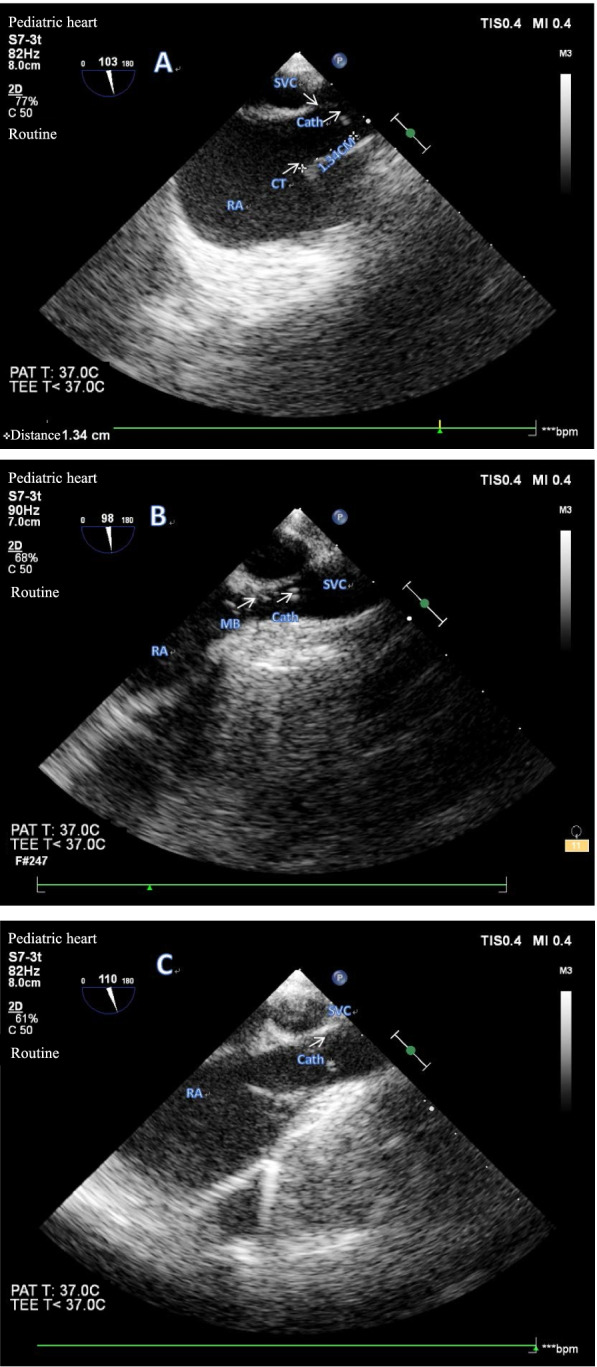
**A** TEE image of the catheter tip finally positioned, directly visualizing the end of the venous catheter 13.4 mm above the crista terminalis. **B** Multiple hyperechogenic microbubbles flowing quickly out of the distal catheter after a rapid flush of saline, confirming the location of the catheter tip. **C** Final confirmation of the location of the catheter tip using TEE after the catheter was connected to the port. SVC, superior vena cava; RA, right atrium; PAT, patient; TEE, transesophageal echocardiography

The Philips pediatric TEE probe (S7-3) covered with a sterile and lubricated protector was gently inserted into the mid-esophagus of the patient after the endotracheal intubation. Then, the probe was rotated to the right, and its angle was adjusted to 90–110° to obtain a mid-esophageal bicaval view, which showed a longitudinal view of the atria and vena cava [14]. After confirming that the catheter was inserted into the right/left IJV, 3 TEE operators observed the presence of the catheter in the right atrium (RA) under real-time TEE visualization. TEE could visualize the catheter by showing parallel hyperechoic lines (double track sign) from the SVC to the RA (Fig. 4B). Then, the catheter was pulled back to ensure the tip was located at the SVC-RA junction (Fig. 4A). The length of the catheter beneath the skin was considered to be equal to the distance from the puncture site to the SVC-RA junction (L1). While the catheter was cut and connected to the port, the right location of the catheter tip would finally be confirmed using TEE (Fig. 4C), which was identified by directly visualizing many hyperechogenic microbubbles quickly flowing out of the distal catheter after a rapid flush of heparin saline [15]. All TEE capturing and image reading were done by a certain member of the research team who specialized in TEE.

### Follow-up

In this study, the endpoint of follow-up was defined as the death of the patient and the end of the 6-month follow-up period or port explantation [16]. After insertion of TIVADs, all the patients were followed up for at least 1 week during hospitalization to evaluate perioperative complications. Perioperative complications refer to complications that occur during surgery or within 24 h after implantation. In the first and sixth months, early and late complications were assessed, respectively. Any complications that occurred during this follow-up period were recorded.

### Data collection

All the patients’ general information, time of surgery, chest radiography images after surgery, and immediate and short-term complications such as bleeding, hematoma, incision dehiscence, catheter blockage or distortion, catheter malposition, incision infection, delayed healing, and port damages were obtained.

### Statistical analysis

The Shapiro–Wilk test was used to check the normal distribution. Measurement data with normal distribution were shown as the mean ± standard deviation (mean ± SD), and those with skewed distribution were described by the median and quartile [median (*Q*_1_, *Q*_3_)]. Enumeration data were expressed as the number of cases and constituent ratio [*n* (%)]. All data were analyzed with NCSS PASS 15.0 (NCSS, Kaysville, UT, USA).

## Results

### Patient characteristics

In this retrospective study, a total of 294 pediatric patients were involved. All the patients’ general information, time of surgery, and type of diseases are recorded in Table 1. Generally, 242 cases had satisfactory catheter tip placement under TEE guidance, with a success rate of 82.3%. The classification of complications was based on the recommendations of the Society of Interventional Radiology (SIR) [17]. A total of 72 complications were recorded. There was one case of a perioperative complication, 66 early complications, and 5 late complications after port implantation. The most common complications were local infection and catheter malposition. Six cases were re-implanted on the left JV due to complications. There were no complications such as bleeding, hematoma, incision dehiscence, catheter blockage or distortion, and delayed healing detected in this study.

**Table 1:** Demographic and clinical characteristics in 294 patients

Characteristics	Total (*n* = 294)
Sex distribution, male:female	172:122
Age (months), median (*Q*_1_, *Q*_3_)	36 (24, 60)
Weight (kg), median (*Q*_1_, *Q*_3_)	14.2 (7.7, 20.7)
Follow-up period (months)	6
Surgery time (min), median (*Q*_1_, *Q*_3_)	64 (47.3, 80)
Diseases, *n* (%)	
Hematological malignancy	199 (67.7%)
Malignant solid tumor	88 (29.9%)
Non-tumor	4 (1.4%)
Immunodeficiency disease	3 (1.0%)

### Perioperative complications

Only one case failed the puncture on the right JV, then catheterization and port implantation were performed on the left side successfully. Overall, no bleeding, hematoma, pneumothorax, or hemothorax was observed during the perioperative period.

### Early complications

Early complications occurring within the first 30 days are summarized in Table 2. Generally, local infection and catheter malposition were the most common complications. Postoperative local infection occurred in 8 cases. Among these, 5 cases did not develop an abscess by cleaning the incision with local or systemic antibiotics and sterile dressing every 1 to 2 days, which was certified to be effective in all of these five cases. One case with an abscess had to be treated with debridement and drainage in the operating room, but the port was not removed. For the other 2 cases, the primary port had to be removed from the infected port chamber and re-implanted a new port on the left side after the infection was controlled since peripherally inserted central catheter (PICC) implantation failed. Catheter malposition was seen in 58 cases. Of 4 cases, 1 case was located at T4, 1 case at T8, and 2 cases at T7. The research team repositioned the catheter tip in the operation room successfully since the catheter tip entered the RA. In 1 case located at T7, TEE confirmed that the catheter tip was located just 0.5 cm under the junction of the SVC and the RA, and no treatment was given after a deliberate discussion with hematologists. The remaining 53 cases were located at T2/T3/T4/T8 without any treatment.

**Table 2:** Complications of TEE-guided TIVAD implantation

Complications	Perioperative (≤ 24 h)	Early (≤ 30 days)	Late (≥ 30 days)	Total
Incision infection				10
Dressing change		5		5
Debridement		1		1
Removed port		2	2	4
Catheter malposition				58
Repositioned		4		
Examination or no treatment		54		
Explantation				6
Re-implanted		2	1	3
PICC or SVC			3	3
CRT			1	1
Port punctured			1	1
Residues			1	1
Venipuncture failure	1			1

*TIVAD* totally implantable venous access device, *TEE* transesophageal echocardiography, *PICC* peripherally inserted central catheter, *SVC* superior vena cava, *CRT* catheter-related thrombosis

### Late complications

The late complications occurring more than 30 days after implantation were recorded in 5 patients. Incision infection occurred in 2 cases. The primary port was removed due to infection, and PICC implantation was performed in one case. The other one suffered from catheter-associated bacteremia, in which the port was removed immediately and a new one was re-implanted on the left side after infection controlled by treatment with approaches to port-related infection [18, 19]. One catheter with fluid leakage was found due to improper use, and a PICC was implanted after removing the damaged port. In one case, a patient with catheter-related thrombosis (CRT) had to get removal of the port and then receive insertion of a left central venous catheter. In one case, an attachment of the port was found in the subcutaneous tissue half a year after the removal of the port, and surgery was performed to remove the attachment.

### Catheter tip position

The proper position in children shown on the chest radiography was between T5 and T7 segments [20]. The results of the final position of the catheter tip on postoperative chest radiography are summarized in Table 3. It shows that 242 catheters were in the proper position with TEE guidance. The positions of the catheter tips in 55 cases were detected out of this range or received a re-implantation, in which 2 cases went beyond T7 and ended at T8, 2 cases ended at T2, 11 cases ended at T3, and 40 cases ended at T4. Postoperative chest radiography found that 3 cases were located at the T7 level, but transthoracic echocardiography (TTE) found that the RA was entered in 2 cases for re-implantation, and TEE examination found that the catheter tip of the other 1 case was just 0.5 cm below the junction without any treatment in the end. Except for the only one case that ended at T4, all the other catheters that ended at T3 and T4 were not adjusted after a discussion with the hematologists, since it did not affect therapy and there were no complications such as arrhythmia and leakage. CRT occurred in one of the two patients whose catheters ended at T2 3 months after implantation, and the other one was untreated with no complications observed. One patient whose catheter tip ended at T8 got repositioning of the catheter tip a few days after primary implantation.

**Table 3:** Distribution of the tip position in the thoracic vertebral plane and coincidence rate of chest X-rays and TEE in 294 patients

Thoracic	Patients, *n* (%)
T2	2 (0.68%)
T3	11 (3.7%)
T4	40 (13.6%)
T5	138 (46.9%)
T6	74 (25.2%)
T7	30 (10.2%)
T8	2 (0.68%)
Coincidence rate	242 (82.3%)

*TEE* transesophageal echocardiography

## Discussion

Various techniques were used to improve the accuracy of TIVAD positions, such as intravenous electrocardiography [9], depth calculation formulas [21], or interventional guidance, followed by postoperative chest radiography for position adjustment [2, 22]. These techniques have deficiencies in port implantation. From June 2018, we tried to accurately implant TIVADs via an internal jugular access under TEE guidance. This retrospective study presented our experiences and analyzed the positions of catheter tips and complications in TEE-guided implantation of TIVADs, involving 294 pediatric patients. A percutaneous approach was adopted in all cases in the right or left IJV following a standard protocol. All the implantation was completed successfully in our study.

There are some contraindications for TIVAD implantation, including chest wall deformity, clavicle fracture, history of head and neck surgery, history of surgical local radiotherapy, bacteremia/sepsis, severe obstructive pulmonary disease, pulmonary embolism, and tumors that cause SVC stenosis. We performed evaluations before port implantation to avoid contraindications. The purpose of port implantation is mainly to inject chemotherapy drugs. Hematological malignancies with low immune function and coagulation function, often accompanied by neutropenia and thrombocytopenia, are indications for TIVAD implantation. We carried out corresponding treatment for these children with hematological malignancies before port implantation, such as platelet transfusion (to reach a target value of 50 × 10^9^/L), improving coagulation function and other measures, to ensure the safety of surgery. In addition, due to the influence of body position and respiration, there is still a certain difference in the position of the catheter tip in different imaging evaluations. Full knowledge of anatomy and imaging is required to evaluate the tip location and availability to avoid re-implanted and secondary adjustments.

In this study, 242 cases were confirmed to be in a good position both by TEE and postoperative radiography, with 82.3% of sensitivity, which was consistent with the study of Yang et al. [12]. The catheter tips of 40 (13.6%) cases were found to be located at T4 on supine chest radiographs. Except for the catheter tip of one case located in RA by TEE and receiving a second repositioning, the other remaining 39 cases did not receive treatments, and no complications were observed. Consistently, the investigation of Yang et al. [12] showed a coincidence rate of 80.56% in correctly detecting the location of the catheter tip on postoperative chest radiography compared with intraoperative TEE assessment. In their study, the remaining 19.44% of children also had the catheter tips located at T4, with no complications and no treatments for these cases. In this case, it seems reasonable for pediatric patients when the catheter tip was located at the range of T4–T7 based on postoperative chest radiography, not just between T5 and T7.

The proper echocardiographic position of the catheter tip was in the SVC within 1 cm of the SVC-RA junction defined as the superior edge of the crista terminalis for pediatric patients of all ages [23]. In the early stage of our study, we also positioned the catheter tip in the SVC within 1 cm of the SVC-RA junction. Then, we found the catheter tips of 3 patients were shown on TEE to be correctly placed in the SVC within 1 cm of the SVC-RA junction intraoperatively but were found to be just below 0.5 cm in the RA by postoperative radiography, which mainly occurred in patients aged 2–4 years with shorter SVC. The research of Andropoulos et al.[15] also demonstrated that 21 of 141 patients whose TEE showed catheters in good positions had apparent positions in the RA on radiographs. In most of them, the catheters were positioned just below the SVC-RA junction. For small infants, the distance from the catheter entrance into the vein to the RA is often ≤ 5 cm, and it is considerable to position the catheter tip in the SVC within 1 cm of the SVC-RA junction as illustrated before [23]. However, the catheter tip position may change from the low SVC initially to the RA with changes from supine to upright positions, and the tip migration was more frequent for older pediatric patients if the catheter tip was located only 1 cm below the SVC-RA junction. In our practice, for older pediatric patients, higher positioning of the catheter tip was above the pericardial reflection and had a wider range of movement, which had minimal risks of the catheter tip entering the RA and the occurrence of direct impingement on the cardiac chamber. At the same time, the catheter tip above the SVC-RA junction for 1–2 cm could be visualized easily using TEE. Accordingly, to correctly position the catheter tip at the SVC-RA junction in pediatric patients of different ages, we intentionally adjusted the insertion depth of the catheter tip later in this study, and no catheters were found to enter the RA anymore. Hence, for patients aged less than 1 year, the catheter tip may be positioned in the SVC within 1 cm of the SVC-RA junction, while for patients aged more than 1 year, the catheter tip position in the SVC within 1–2 cm of the SVC-RA junction seems more reasonable.

We also found that there were still cases in which postoperative chest radiography and intraoperative TEE showed differences in the position of the catheter tip. Possible reasons for the above events are as follows: (1) Inconsistency of the head position between intraoperative TEE and postoperative chest radiography results in the catheter tip moving backward above T4 and forward into the atrium in the head backward and forward flexion positions, respectively. Moreover, chest radiography can be affected by anatomical location and breathing. (2) The appropriate position of TIVAD catheterization is different for TEE and chest radiography. The radiographic SVC-RA junction was defined as the apex of the concave shadow formed by the superimposition of the distal SVC on the RA [24], while the echocardiographic SVC-RA junction was defined as the superior edge of the crista terminalis [25]. (3) The location of the heart varies among human beings. It can be transverse, vertical, or even oblique anatomically, resulting in different projections of the heart on the spine segment. (4) Operating error caused by the operators when establishing the subcutaneous tunnel from the venipuncture point to the port chamber may lead to inaccuracy of the final length of the catheter. These cases mainly occurred in the first half year when the research group started using TEE for guidance, and these were significantly reduced as the research progressed. Therefore, operators and radiologists should discuss and develop a standard protocol of patient position when using chest radiography to confirm the position of the catheter tip, so as to reduce the likelihood of reposition due to the inconsistent depth caused by different head positions.

A total of 10 cases of infection occurred in this study, with an incidence of 10/297 (3.4%). Catheter-related infection is defined as before [26], which is divided into several types such as port infection, exit site infection, bacteremia, and catheter-related sepsis. Infection is a serious complication for patients and remains to be a challenging issue in TIVAD implantation, especially for those patients with hematological cancers receiving chemotherapy, which is consistent with the findings of Wang et al. [27] in Chinese cancer patients. Suffering from infection reduces the quality of medical care and increases the healthcare costs of patients. Strengthened incision nursing should be performed to avoid postoperative infection. At the same time, increasing the number of white blood cells may improve the immunity of cancer patients. Previous studies have shown that the incidence of intraoperative TEE-related complications is 0.2–1.2%, and the mortality rate is 0% [28]. In this study, we did not find TEE-related complications that required targeted treatment, such as oropharyngeal esophageal mucosal damage, bleeding, sore throat, and dysphagia so on, indicating that the use of TEE was safe. The reasons may be the following: (1) The age of pediatric patients was more than 3 months, and a probe suitable for young children was chosen; standardized operations were strictly carried out to avoid blindly sending the probe and used the laryngoscope to assist if necessary. (2) The esophagus ultrasound protector was used and fully lubricated, and the operation was meticulous and gentle, which avoided the occurrence of esophageal mucosal damage. (3) The esophageal ultrasound probe was placed for a short time, usually about half an hour, and the mucosa was compressed for a short time, and no pressure injury was formed. Besides, the catheter location was as expected in more than 80% of cases, reducing the occurrence of CRT to a certain extent, and CRT was found in only one case at T2. CRT in this case was located where the catheter entered the IJV, attached to the inner wall of the blood vessel, and organized with the blood vessel wall. The position and shape of CRT did not change significantly after the catheter was removed. The formation of this CRT may be related to the vascular intimal injury resulting from puncture and not be related to the position of the catheter tip.

When comparing intraoperative TEE with postoperative radiography, TEE has more significant advantages. Chest radiography performed postoperatively may possibly alter the position of the catheter tip after surgical dissection and manipulation. In addition, the catheter is in a dynamic system which can be affected by the changes in postures and diaphragmatic movements. In contrast, the TEE technique positively identifies catheter tip location intraoperatively in the SVC by direct visualization of the vessel. With TEE, adjustments to the catheter position can be made at the time of placement, rather than postoperatively. Moreover, it seems that the SVC-RA junction can be more precisely determined under real-time TEE visualization intraoperatively than by a supine chest radiograph postoperatively. Other prior studies also demonstrated that TEE provided a more sensitive assessment than chest radiography [10, 15]. Based on the above advantages, we believe that it is more suitable for pediatric patients to apply TEE to confirm the proper position of the catheter tip in TIVAD implantation, and TEE significantly improves the success rate of proper placement. The use of chest radiography to confirm the catheter position may need to be reassessed.

There are several limitations of our study. The results from TEE and chest radiography were not directly compared, and we did not compare TEE with fluoroscopy and TEE with TTE. In clinical practice, corresponding anatomical structures can be observed through TEE during the operation, and the tip of the infusion tube can be positioned and adjusted in real time under direct vision [29]. TEE is low-cost and radiation-free. In contrast, the intracardiac structure cannot be seen under intraoperative fluoroscopy, and the relationship between the atrium and the superior vena cava can only be roughly determined. The image resolution of fluoroscopy is not high, and it is difficult to clearly distinguish some parts with relatively large density or thickness, but fluoroscopy is easy to operate. As regards TTE, it is non-invasive, but its image quality may be insufficient [30]. Future studies are warranted for these comparisons in pediatric patients, and we are currently conducting research on the comparison between TEE and TTE in the implantation of TIVADs. Besides, using TEE, the tip of the catheter is sometimes difficult to distinguish from other intracardiac structures, such as the Eustachian valve and the SVC wall. Thus, operators are required to master the TEE and cardiac anatomy; otherwise, the accuracy of catheter tip positioning may be reduced. Furthermore, 17 cases (5.7%) were lost to follow-up because this study focused on short-term follow-up, and the results of long-term follow-up need to be explored.

## Conclusion

TEE was accurate and safe in confirming the proper position of the TIVAD catheter via the IJV for pediatric patients, which may act as a reference method to locate the TIVAD catheter.

## Data Availability

The datasets used and/or analyzed during the current study are available from the corresponding author on reasonable request.

## Abbreviations

TIVADs: Totally implantable venous access devices
TEE: Transesophageal echocardiography
TTE: Transthoracic echocardiography
CRT: Catheter-related thrombosis
PICC: Peripherally inserted central catheter
VT: Tidal volume
RR: Respiratory rate
ETCO_2_: End-tidal CO_2_
ECG: Electrocardiogram
IJV: Internal jugular vein
SVC-RA: Superior vena cava-right atrial
PACU: Post-anesthesia care unit
SD: Standard deviation
SIR: Society of Interventional Radiology

## References

[1] Pinelli F, Cecero E, Selmi V, Giua R, Villa G, Degl’Innocenti D (2018). Infection of totally implantable venous access devices: a review of the literature. J Vasc Access..

[2] Kao CY, Fu CH, Cheng YC, Chen JL, Cheng YC, Chen CC (2020). Outcome analysis in 270 radiologically guided implantations of totally implantable venous access ports via basilic vein. J Chin Med Assoc.

[3] Kim JT, Oh TY, Chang WH, Jeong YK (2012). Clinical review and analysis of complications of totally implantable venous access devices for chemotherapy. Med Oncol.

[4] Yazıcı N, Akyüz C, Yalçın B, Varan A, Kutluk T, Büyükpamukçu M (2013). Infectious complications and conservative treatment of totally implantable venous access devices in children with cancer. Turk J Pediatr.

[5] Wang C (2014). Application and nursing of venous port access in pediatric cancer patients. J Chin Physician.

[6] Liu B, Xue Y, Zhu H, Pei Y, Si R, Song R (2019). Clinical application and nursing research of totally implantable venous access ports in children with hematological tumors. Shanxi Med J..

[7] Uchida Y, Sakamoto M, Takahashi H, Matsuo Y, Funahashi H, Sasano H (2011). Optimal prediction of the central venous catheter insertion depth on a routine chest x-ray. Nutrition (Burbank, Los Angeles County, Calif).

[8] Zhou C, Lu L, Yang L, Xi W, Ma T, Yang C (2021). Modified surface measurement method to determine catheter tip position of totally implantable venous access port through right subclavian vein. J Vasc Surg Venous Lymphat Disord.

[9] Wang YC, Huang CH, Lin FS, Lin WY, Fan SZ, Lin CP (2012). Intravenous electrocardiography helps inexperienced operators to place totally implantable venous access device more accurately. J Surg Oncol.

[10] Reynolds N, McCulloch AS, Pennington CR, MacFadyen RJ (2001). Assessment of distal tip position of long-term central venous feeding catheters using transesophageal echocardiology. JPEN J Parenter Enteral Nutr.

[11] Witthayapraphakorn L, Khositseth A, Jiraviwatana T, Siripornpitak S, Pornkul R, Anantasit N (2013). Appropriate length and position of the central venous catheter insertion via right internal jugular vein in children. Indian Pediatr.

[12] Yang S, Kong X, Liu L, Xu Y, Zhang J (2021). Application of transesophageal echocardiography for localization in totally implantable venous access port implantation through subclavian approach in children. Clin Cardiol.

[13] Bajaj M, Offiah AC (2015). Imaging in suspected child abuse: necessity or radiation hazard?. Arch Dis Child.

[14] Hahn RT, Abraham T, Adams MS, Bruce CJ, Glas KE, Lang RM (2014). Guidelines for performing a comprehensive transesophageal echocardiographic examination: recommendations from the American Society of Echocardiography and the Society of Cardiovascular Anesthesiologists. Anesth Analg.

[15] Andropoulos DB, Stayer SA, Bent ST, Campos CJ, Bezold LI, Alvarez M (1999). A controlled study of transesophageal echocardiography to guide central venous catheter placement in congenital heart surgery patients. Anesth Analg.

[16] Teichgräber UK, Kausche S, Nagel SN, Gebauer B (2011). Outcome analysis in 3,160 implantations of radiologically guided placements of totally implantable central venous port systems. Eur Radiol.

[17] Madan M, Shah MV, Alexander DJ, Taylor C, McMahon MJ (1994). Right atrial electrocardiography: a technique for the placement of central venous catheters for chemotherapy or intravenous nutrition. Br J Surg.

[18] Mermel LA, Allon M, Bouza E, Craven DE, Flynn P, O’Grady NP (2009). Clinical practice guidelines for the diagnosis and management of intravascular catheter-related infection: 2009 update by the Infectious Diseases Society of America. Clin Infect Dis.

[19] Tabatabaie O, Kasumova GG, Eskander MF, Critchlow JF, Tawa NE, Tseng JF (2017). Totally implantable venous access devices: a review of complications and management strategies. Am J Clin Oncol.

[20] Connolly B, Mawson JB, MacDonald CE, Chait P, Mikailian H (2000). Fluoroscopic landmark for SVC-RA junction for central venous catheter placement in children. Pediatr Radiol.

[21] Cleff C, Boensch M, Eifinger F (2018). Hinkelbein J. Der [Correct positioning of central venous catheters in pediatrics: are current formulae really useful?]. Anaesthesist.

[22] Wright D, Williams D (2020). Central venous catheter tip position on chest radiographs. Anaesthesia.

[23] Chu KS, Hsu JH, Wang SS, Tang CS, Cheng KI, Wang CK (2004). Accurate central venous port-A catheter placement: intravenous electrocardiography and surface landmark techniques compared by using transesophageal echocardiography. Anesth Analg.

[24] John SD. SL. Differential diagnosis in pediatric radiology. 2nd ed. Baltimore: Williams & Wilkins; 1995.

[25] NH. S. Pediatric echocardiography. Baltimore: Williams & Wilkins; 1993.

[26] Silberzweig JE, Sacks D, Khorsandi AS, Bakal CW (2003). Reporting standards for central venous access. J Vasc Interv Radiol.

[27] Wang TY, Lee KD, Chen PT, Chen MC, Chen YY, Huang CE (2015). Incidence and risk factors for central venous access port-related infection in Chinese cancer patients. J Formos Med Assoc..

[28] Hahn RT, Abraham T, Adams MS, Bruce CJ, Glas KE, Lang RM (2013). Guidelines for performing a comprehensive transesophageal echocardiographic examination: recommendations from the American Society of Echocardiography and the Society of Cardiovascular Anesthesiologists. J Am Soc Echocardiogr.

[29] Corradi F, Guarracino F, Santori G, Brusasco C, Tavazzi G, Via G (2022). Ultrasound localization of central vein catheter tip by contrast-enhanced transthoracic ultrasonography: a comparison study with trans-esophageal echocardiography. Crit Care.

[30] George JC, Varghese V, Mogtader A (2014). Intracardiac echocardiography: evolving use in interventional cardiology. J Ultrasound Med..

